# The Immunomodulatory Effect of Radiofrequency Electromagnetic Field on Serum Cytokine Levels in A Mouse Model of Hindlimb Unloading

**DOI:** 10.22074/cellj.2021.6856

**Published:** 2020-04-22

**Authors:** Sima Aghajari, Sayed Mohammad Javad Mortazavi, Mehdi Kalani, Samaneh Nematolahi, Parham Habibzadeh, Shirin Farjadian

**Affiliations:** 1. Department of Radiology, Shiraz University of Medical Sciences, Shiraz, Iran; 2. Department of Medical Physics, Shiraz University of Medical Sciences, Shiraz, Iran; 3. Department of Immunology, Shiraz University of Medical Sciences, Shiraz, Iran; 4. Professor Alborzi Clinical Microbiology Research Center, Shiraz University of Medical Sciences, Shiraz, Iran; 5. Department of Biostatistics, Shiraz University of Medical Sciences, Shiraz, Iran; 6. R&D Department, Persian BayanGene Research and Training Center, Shiraz, Iran; 7. Student Research Committee, Shiraz University of Medical Sciences, Shiraz, Iran

**Keywords:** Cytokine, Flow Cytometry, Hindlimb Suspension, Radio Wave

## Abstract

**Objective:**

Astronauts are exposed to a wide range of environmental stresses during spaceflights that reduce their immune responses and make them more susceptible to infections and malignancies. Exposure to a low dose of a certain stress induces an adaptive response, which leads to resistance to higher doses of the same or other types of stress. We designed this study to investigate the effect of radiofrequency electromagnetic field (RF-EMF)-induced adaptive response on immune system modulation in a mouse model of hindlimb unloading (HU) as a ground-based animal model of spaceflight conditions.

**Materials and Methods:**

In this experimental study, serum levels of T helper (Th)-mediated cytokines were determined by the multiplex cytometric bead assay in four groups of mice (n=10 per group): HU mice, RF-EMF-treated mice, HU mice pre-exposed to RF-EMF; and untreated controls. Mice were exposed to 2450 MHz RF-EMF with SAR 0.478 W/ kg for 12 hours/day for three successive days.

**Results:**

Tumor necrosis factor-alpha (TNF-α), interleukin-9 (IL-9) and IL-22 were significantly decreased in HU mice. Comparison between HU mice and RF-EMF-treated mice showed an opposite change in IL-6, while IL-9, IL-22, IFN-γ and TNF-α decreased in both groups. However, just interferon gamma (IFN-γ) was significantly decreased in HU mice that were pre-exposed to RF-EMF compared to the control group.

**Conclusion:**

The effect of RF-EMF in elevating IL-6 and reducing IL-9 in opposite directions in HU mice suggest a modulating effect of RF-EMF on HU-induced changes in these cytokines, as Th2 and Th9 eventually returned to normal levels and balances in cytokine ratios were also restored in HU mice pre-exposed to RF-EMF.

## Introduction

During spaceflight, astronauts encounter a variety of environmental changes (1) such as microgravity (2) and exposure to radiation and solar energetic particles (3, 4). Along with circadian rhythm disturbances (5) and altered nutritional intake (6), these changes may lead to dysregulation of physiological functions. Impaired immune responses to infectious agents and malignant cells may be life-threatening to space travelers (7, 8).

The fine-tuning of immune responses is mediated by cytokines secreted mainly by T helper (Th) cells. While Th2-mediated humoral immunity plays a major role against extracellular pathogens, cellular immunity mediated by Th1 cells acts as an essential response to viruses and tumor cells. Furthermore, Th17 cells contribute to the clearance of extracellular microorganisms by neutrophilic inflammation. These cells also promote mucosal and epithelial barrier functions. Th9 is crucial for defense against helminthes, and Th22 cells found mainly in the epidermis play an important role in chronic inflammatory skin disorders (9).

There is some evidence of immune deregulation during extended space missions (10, 11). Spaceflight represents a unique situation that results in numerous changes in the human body. The study of immune reactivity before, during and after brief or extended flights is essential for understanding integrated responses in the complex environment that astronauts inhabit. Since many experiments cannot be performed in these conditions, ground-based models that simulate spaceflight conditions can help take this research forward. Mouse models of hindlimb unloading (HU) are widely used to mimic the effect of microgravity during spaceflight on mouse physiology (12).

Adaptive response is the exposure to a low dose of a certain stress that leads to resistance to higher doses of the same or other types of stress (13). Adaptive response was first reported by Samson and Cairns (14) in 1977 when they observed bacterial resistance to a high dose of an alkylating mutagen following bacterial growth in a nontoxic dose of the same substance. In 1984, Olivieri et al. (15) also found that human lymphocytes exposed to 3H-thymidine, as a source of lowlevel chronic radiation, became more resistant to chromosomal aberration that resulted from high doses of X-rays. It was previously shown that laboratory animals pre-exposed to a radiofrequency electromagnetic field (RF-EMF) were more resistant to subsequent high doses of ionizing radiation or infections caused by life-threatening microorganisms (16- 18). Zeni et al. (19) observed a remarkable decrease in the frequency of micronuclei formation in lymphocytes of individuals who were pre-exposed to 1950 MHz RF-EMF at a specific absorption rate (SAR) of 0.3 W/kg for 20 hours and then challenged with mitomycin C. Jiang et al. (20, 21) observed a notable reduction in DNA damage in blood and bone marrow leukocytes of mice that were pre-exposed to an adaptation dose of 900 MHz RF-EMF at a power density of 120 mW/cm2 for 4 hours/day for 3-14 consecutive days, and then exposed to 3 Gy whole-body γ-radiation. In the current study, we compared serum cytokine levels in HU mice with and without RF-EMF-treatment to untreated mice in order to investigate the effects RF-EMF-induced adaptive response on immunomodulation in microgravity conditions.

## Materials and Methods

### Study design

In this experimental study, 6-week-old male BALB/c mice with a mean body weight of 25-30 g were housed under controlled conditions at a temperature of 23 ± 1˚C, humidity of 50 ± 5% and equal light/dark cycle. The experimental protocols were approved by the Ethics Committee of Shiraz University of Medical Sciences (approval code: IR.SUMS. REC.1394.S59) based on the "Guide for the Care and Use of Laboratory Animals" published by the National Academy Press (22).

After a 7-day isolation period, the animals were randomly allocated to four groups (10 mice per group): untreated mice (G1), mice with HU (G2), RF-EMF-treated mice (G3) and HU mice that were pre-exposed to RF-EMF (G4). Blood samples were collected from each mouse 24 hours after the last intervention in each group. All serum samples were isolated and stored at -20˚C until further use.

### Hindlimb unloading mouse model

Hindlimb unloaded mice were prepared as previously described (23). Briefly, one week after inserting a stainless steel ring between the L5 and L6 mouse vertebrae, the tail ring was connected to a bobbin in a rail mounted at the roof of a plastic cage using an S-shaped hook. Each mouse was suspended by the tail with a 20-degree angle of hind limbs to the horizon. During this time, the animals had free access to food and water.

### Radiofrequency irradiation

An AD-link Wi-Fi router was used as the source of RF-EMF. During the exposure period, data was shared between the WiFi router and a laptop at a distance of 6 m in an adjoining room. The Wi-Fi router operated at a power level of 1 W and the device was located 30 cm from the animals’ cage. Mice were exposed to 2450 MHz RF-EMF at SAR 0.478 W/kg for 12 hours/day for 3 successive days. All experiments were performed in an environment with a negligible background level of electric and magnetic fields.

### Cytokine assay

Serum levels of Th-related cytokines that included Th1 (IFN-γ, TNF-α and IL-2), Th2 (IL-4, IL-5, IL-6, IL-10 and IL- 13), Th17 (IL-17A, IL-17F and IL-21), Th9 (IL-9) and Th22 (IL-22) were quantified with a multiplex cytometric bead assay using a commercial kit (BioLegend, San Diego, CA, USA) according to the manufacturer’s directions. Briefly, a mixture of FITC-labeled antibody-coated beads for each desired cytokine, which could be differentiated by their sizes and fluorescence intensities, was incubated with the mouse serum samples or standards. After capturing the cytokines by the beads, biotin-conjugated anti-mouse antibody and PElabeled streptavidin were successively added. The results were visualized with a FACSCalibur flow cytometer (eBioscience, San Diego, CA, USA) and the data were analyzed with FlowCytomix Pro-3.0 software (BioLegend).

### Statistical analysis

The Shapiro-Wilk test was used to verify normal distribution of the data. The nonparametric Kruskal-Wallis test was used to compare cytokine levels among groups. Then, post hoc pairwise multiple comparisons were performed with Dunn’s test. All statistical analyses were done with SPSS 23 (SPSS Inc., Chicago, Illinois, USA) and a two-sided P≤0.05 was considered statistically significant. GraphPad Prism 6.0 (GraphPad Software Inc., La Jolla, San Jose, CA, USA) was used to generate the graphs.

## Results

We investigated the effect of RF-EMF-induced adaptive response on the immune system in HU mice. To this effect, serum levels of Th-related cytokines were determined in HU mice, RF-EMF-treated mice and HU mice that were preexposed to RF-EMF in comparison to untreated mice.

**Fig.1:**
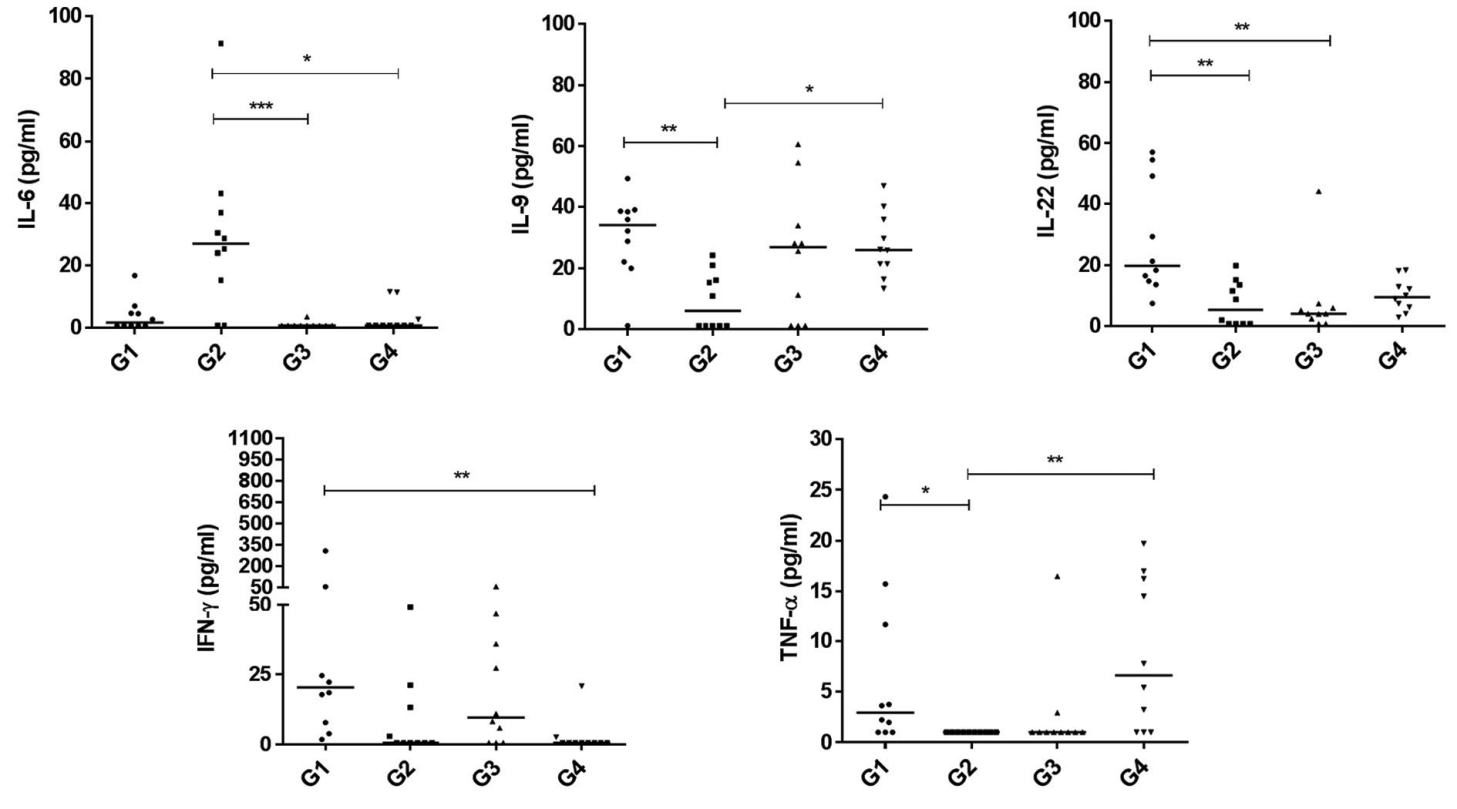
Comparison of cytokine serum levels among four mouse groups (n=10 in each group). Mouse groups are G1; Untreated mice, G2; Hindlimb unloading (HU) mice, G3; Radiofrequency electromagnetic field (RF-EMF)-treated mice, and G4; HU mice that were pre-exposed to RF-EMF. *; P<0.05, **; P<0.01, and ***; P<0.001.

Figure 1 shows the significant changes in cytokine levels among the studied groups. As shown, there was a decrease in IL-9 (P=0.007), IL-22 (P=0.006), TNF-α (P=0.029) and IFN-γ (non-significant, NS) levels, whereas IL-6 (NS) levels increased in HU mice compared with the control group (G2 vs. G1). A comparison of RF-EMF-treated mice to the control group (G3 vs. G1) showed an increase in IL-9 (NS) and decrease in IL-22 (P=0.001), TNF-α (NS), IFN-γ (NS) and IL-6 (NS) levels. A comparison between HU mice and RF-EMF-treated mice showed the opposite, an increase in IL-6 (0.001), whereas IL-9, IL-22, IFN-γ and TNF-α levels decreased in both groups. However, only IFN-γ had a significant decrease in HU mice that were pre-exposed to RFEMF compared with the control group (G4 vs. G1).

**Fig.2:**
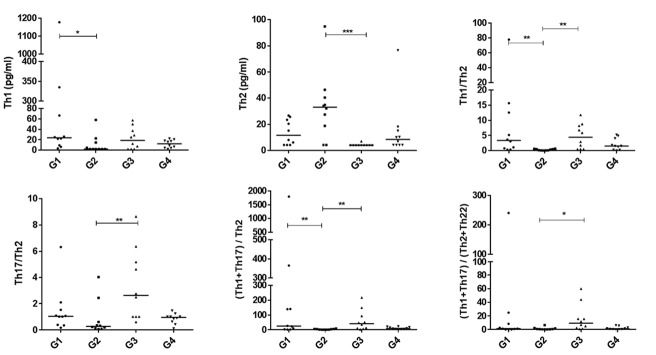
Comparison of cytokines in each T helper (Th) cell subset and their ratios among four mouse groups (n=10 in each group). Mouse groups are G1; Untreated mice, G2; Hindlimb unloading (HU) mice, G3; Radiofrequency electromagnetic field (RF-EMF)-treated mice, and G4; HU mice that were pre-exposed to RF-EMF. *; P<0.05, **; P<0.01, and ***; P<0.001

Figure 2 shows the cytokine changes in Th subsets and their ratios. Th1 levels significantly decreased (P=0.033), Th2 slightly increased (NS), and the Th1/Th2 ratio decreased significantly (P=0.008) in G2 compared to G1 mice. Although Th17 showed no change between these two groups, the (Th1+Th17)/Th2 ratio (P=0.009) was significantly decreased in G2 compared to G1. Th subsets and their ratios showed no remarkable differences between G3 compared to G1. However, significant changes, in the opposite directions, were observed in Th2 (P=0.001), Th1/Th2 (P=0.006), Th17/Th2 (P=0.003), (Th1+Th17)/Th2 (P=0.002) and (Th1+Th17)/ (Th2+Th22) (P=0.002) between G2 and G3.

## Discussion

We investigated the modulating effect of RF-EMF on HUinduced changes in Th-mediated cytokines by comparing serum cytokine levels in HU mice with and without RF-EMF treatment to untreated mice. Our results showed markedly decreased Th1 levels in HU mice in light of the reduction in IFN-γ and TNF-α. Reactivation of latent viruses in astronauts during long-term spaceflight has previously been reported (24, 25) which might be explained by reduced Th1 responses, although the importance of antibodies in the control of viral infections should not be ignored. In this connection, Gaignier et al. (26) also reported decreased numbers of B cells in the spleen of HU mice and an impaired proliferative response in these cells after mitogen stimulation. However, they used Th1-biased C57BL/6 mice in their experiments instead of the Th2-prone BALB/c mice that we used in the current study (27).

Our results showed a slight increase in Th2 cytokine levels in HU mice, which might be explained by the slight elevation of IL-6. This finding agreed with the results of Jang et al. who found slight change in Th2 cytokines after *in vitro* stimulation of T cells from HU BALB/c mice (28). We found no change in Th17 cytokines in HU mice, which was in line with the results reported by Gaignier et al. (26). In our study, IL-22 levels markedly decreased in HU mice. Although there was no study that directly focused on changes in IL-22 levels in HU mice, Li et al. (29, 30) reported delayed corneal epithelial wound healing in HU mice, which they attributed to decreased levels of IL-22.

In our study, RF-EMF had no crucial effect on IL-9 as well as Th1-, Th2-, and Th17-mediated cytokines; however, there was a strongly decreased IL-22 level in G3 mice compared to the control group.

The opposite changes of IL-6 in G2 compared to G3 mice suggest a modulating effect of RF-EMF on HU-induced changes in this cytokine. This compensatory effect was also observed in IL-9, as eventually Th2 and Th9 returned to normal levels in G4 mice. The modulating effect of RF-EMF on key cytokines might explain the restoration of Th1/Th2, Th17/Th2, (Th1+Th17)/Th2, (Th1+Th17)/ (Th2+Th22) and (Th1+Th17)/(Th2+Th9+Th22) balances in G4 mice.

However, concurrent reduction of IFN-γ, TNF-α and IL-22 was observed following HU induction and after RF-EMF treatment in G2 and G3 mice, respectively. The synergistic effect of both conditions was just detected in IFN-γ, which significantly decreased in G4 compared to the control group.

If further experiments in humans confirm the modulatory effect of RF-EMF on microgravity-induced cytokine changes, this method could be used in future long-term crewed space flights, especially journeys to Mars which are planned for the next decade. Due to similarities between space field complications and prolonged headdown bed rest patients, RF-EMF might also be helpful in immunomodulation of these patients.

## Conclusion

The effect of RF-EMF in elevating IL-6 and reducing IL-9 in opposite directions in HU mice suggests a modulating effect of RF-EMF on HU-induced changes in these cytokines, as Th2 and Th9 eventually returned to normal levels and balances in cytokine ratios were also restored in HU mice pre-exposed to RF-EMF.
